# Tetranuclear Copper Cluster of N_2_O Reductase: A Distinctive Copper–Sulfur Metallocluster

**DOI:** 10.1021/acs.inorgchem.6c04238

**Published:** 2026-10-06

**Authors:** Sofia R. Pauleta, José J. G. Moura, Isabel Moura

**Affiliations:** † Microbial Stress Lab, UCIBIO − Applied Molecular Biosciences Unit, Departamento de Química, 119482 Faculdade de Ciências e Tecnologia, Universidade NOVA de Lisboa, 2829-516 Caparica, Portugal; ‡ Associate Laboratory i4HB - Institute for Health and Bioeconomy, Faculdade de Ciências e Tecnologia, Universidade NOVA de Lisboa, 2829-516 Caparica, Portugal; § LAQV, REQUIMTE, Departamento de Química, Faculdade de Ciências e Tecnologia, Universidade NOVA de Lisboa, 2829-516 Caparica, Portugal

## Abstract

Copper is an essential element involved in several cofactors and organometallic compounds used as catalysts. Although most biological copper centers contain one to three copper ions, nature has evolved a unique tetranuclear copper–sulfur cluster that has attracted the attention of both biochemists and inorganic chemists. This multinuclear copper center has only been observed in one enzyme, nitrous oxide reductase, which catalyzes the reduction of nitrous oxide to dinitrogen, the final step of the denitrification pathway. The structural, spectroscopic, and kinetic properties of this [4CuS] cluster have been the subject of extensive investigation, revealing that it exists in two distinct forms within the enzyme, CuZ­[4Cu:2S] and CuZ*­[4Cu:1S], with different oxidation states, spectroscopic signatures, and catalytic properties. These features are discussed together with recent advances in understanding the biosynthesis and assembly of this cluster. Inorganic chemists have synthesized a variety of structural and functional models of the CuZ cluster, although none fully reproduces the electronic structure and catalytic efficiency of the native active center. This review discusses the interplay between biological and synthetic studies, illustrating how advances in one field continue to inspire progress in the other while identifying the challenges that must be overcome to achieve truly functional biomimetic catalysts.

## Graphical abstract

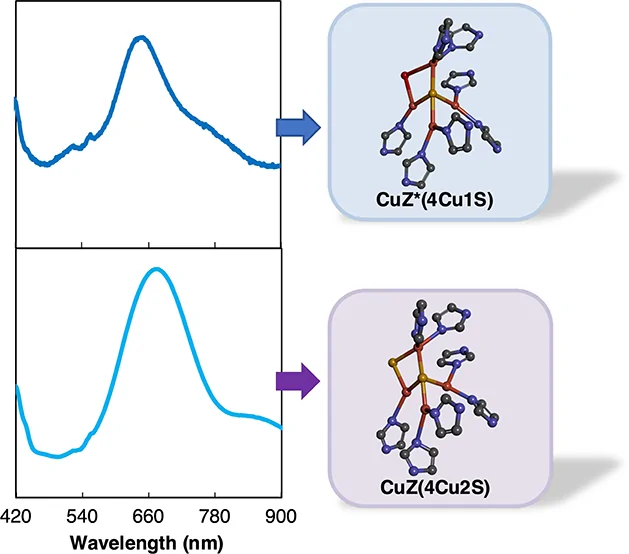

## Introduction

In the expanding universe of biological metal cofactors, true outliers are rare. Most fall into recognizable families, echoing established structural motifs and familiar redox logic. Among biological metallocenters, few have inspired as much fascination as the catalytic center of nitrous oxide reductase (N_2_OR). This enzyme performs the final step of the denitrification pathway, reducing nitrous oxide to dinitrogen, thereby closing the global nitrogen cycle and mitigating emissions of one of the most potent greenhouse gases that also contributes to stratospheric ozone depletion.^1,2^ At the heart of this chemically demanding transformation lies a metal cluster whose structural and electronic features remain without true parallel in either biology or inorganic chemistry.

The active site of N_2_OR, known as the CuZ cluster, contains a unique tetranuclear copper core bridged by a μ_4_-sulfido ligand. This [4CuS] cluster stands apart from the more common mono-, di-, or trinuclear copper centers found in other copper proteins.^3^ Its architecture enforces close metal–metal proximities, extensive electronic coupling, and a level of redox complexity that challenges conventional descriptions based on localized oxidation states. Indeed, the cluster behaves as a highly delocalized redox ensemble, capable of accessing different oxidation states, some of which are catalytically competent while others correspond to inactive or resting states.

What makes this metal cluster truly “unicorn-like” (*rare, difficult to find, much like the mythical creature*) is not merely its nuclearity, but the convergence of several rare properties in a single biological motif. First, the μ_4_-sulfido bridge, unusual in copper biochemistry, plays both structural and electronic roles, mediating coupling between the four copper ions. Second, the cluster exhibits rich redox chemistry, traversing multiple electron-count manifolds with subtle geometric adjustments rather than dramatic structural rearrangements, but depicting subtle control of electron distribution within the core. Third, substrate activation - cleavage of the remarkably inert N–O bond in N_2_O - appears to rely on a stepwise one-electron processes at a single metal cluster.

Despite decades of effort, no synthetic model compound has successfully replicated the full structural, electronic, and functional features of the CuZ cluster. While tetranuclear copper-sulfide clusters can be assembled in the laboratory, reproducing the delicate balance of redox flexibility, stability, and reactivity observed in the protein matrix has proven elusive. This gap highlights not only the sophistication of biological design but also the limitations of current strategies in biomimetic inorganic chemistry.

The CuZ cluster therefore occupies a singular position at the interface of bioinorganic chemistry, catalysis, and environmental science. It challenges our understanding of mixed-valence systems, cooperative multimetallic reactivity, and small-molecule activation. Moreover, it underscores how protein environments can stabilize particular structures, electronic states and reactivity patterns that remain inaccessible in purely synthetic frameworks.

In this review, we examine the structural evolution of the CuZ cluster across oxidation states, the spectroscopic insights that have shaped current electronic descriptions, mechanistic proposals for N_2_O reduction, and ongoing efforts toward functional modeling. By integrating advances from crystallography, spectroscopy, theory, and inorganic chemistry, we aim to highlight why this tetranuclear copper-sulfide cluster remains one of the most extraordinary, and still incompletely understood metallocofactors in biology.

## Discovery of N_2_OR and CuZ Cluster

N_2_OR catalyzes the two-electron, two-proton reduction of nitrous oxide to dinitrogen and water, corresponding to the terminal reaction of denitrification, according to eq 1

$$N_{2}O+2H^{+}+2e^{-}\to N_{2}+H_{2}O,\Delta G^{{}^{\circ}\prime }=-339.5\;\mathrm{kJ}/\mathrm{mol}$$ 1

This reduction is highly exergonic (Δ*G*°′ = −339.5 kJ mol^–1^),^4^ and N_2_O is a strong oxidant with a standard reduction potential of +1.35 V at pH 7.0. Nevertheless, despite this favorable thermodynamic driving force, its reduction is kinetically challenging because of the high activation energy (∼250 kJ mol^–1^),^5^ a consequence of the spin-forbidden nature of the reaction.^6^

The search for N_2_OR started in the early 1970′s, after N_2_O had been recognized as an intermediate of denitrification,^7^ and early physiology studies showed that cells grown anaerobically with N_2_O as the terminal electron acceptor had high N_2_O-reducing activity, indicating the existence of a dedicated enzymatic system for N_2_O respiration^8^ but the enzyme responsible for it remained unknown. In 1972, a bluish-pink copper-containing protein with unique spectroscopic features (absorption bands at 480, 540, 640, and 800 nm) was isolated from *Alcaligenes faecalis*, although neither its function nor enzymatic activity was known.^9^ Ten years later, a protein presenting a similar visible spectrum was isolated from *Pseudomonas stutzeri* ZoBell (classified at the time as *Pseudomonas perfectomarinus*) and its enzymatic activity as nitrous oxide reductase was identified.^10–12^

During the following decade, homologous enzymes were purified from numerous denitrifying bacteria, including *Rhodobacter capsulatus*,^13^ *Paracoccus denitrificans*,^14^ *Achromobacter cycloclastes*,^15,16^ *Pseudomonas aeruginosa*,^17^ *Flexibacter canadensis*,^18^ *Paracoccus* *pantotrophus*,^19^ *Achromobacter xylosoxidans*,^20^ *Thiobacillus denitrificans*,^21^ *Marinobacter nauticus* (formerly named *Marinobacter hydrocarbonoclasticus* or *Pseudomonas nautica*),^22^ *Hyphomicrobium denitrificans* ^23^ and *Wolinella succinogenes*.^24^ Although these enzymes exhibited similar catalytic activities, significant differences were reported in their visible absorption and EPR spectra, copper stoichiometries and specific activities. At the time these differences were generally attributed to species-dependent variations or differences in the purification procedures (anoxic versus oxic).^25–27^ It is now recognized that many of these discrepancies arose from isolation of the enzyme in distinct oxidation states and structural forms of the catalytic copper center, CuZ center, an issue discussed in next sections.

The first indication that N_2_OR contained two spectroscopically distinct copper centers emerged from combined optical, EPR and redox studies during the late 1980s and 1990s. One center exhibited spectroscopic properties remarkably similar to the binuclear mixed-valence CuA center of cytochrome *c* oxidase.^28^ In the oxidized state, this center has absorption bands with maxima at 480 nm (4.0 mM^–1^cm^–1^), 540 nm (4.0 mM^–1^cm^–1^) and 780 nm (3.0 mM^–1^cm^–1^) and displayed the characteristic axial EPR signal with *g*_∥_ = 2.18 and g_⊥_ ∼ 2.02 with seven-line hyperfine coupling (*A*_∥_ = 38 G), due to the unpaired electron delocalization over two equivalent copper nuclei, with a nuclear spin of *I* = 3/2, which explained the well-resolved hyperfine coupling lines (Figure 1A).^29–32^ Structurally it was proposed to be a binuclear copper center with two bridging cysteine thiolate ligands and two terminal histidine nitrogen ligands as CuA center of cytochrome *c* oxidase, in which the small hyperfine coupling constant indicates a high covalent delocalization of the unpaired electron onto the cysteine thiolate ligands (26% S character determined for the CuA center of cytochrome c oxidase by S K-edge X-ray absorption spectroscopy (XAS)).^33^ The similarity between these two copper sites was established by comparison of the room- and low-temperature MCD spectra of N_2_OR and cytochrome *c* oxidase,^30,34^ while the coordination was supported by sequence alignments^35–37^ and site-directed mutagenesis studies,^38^ and subsequently confirmed by the X-ray structure of N_2_OR.^39^

**Figure 1:**
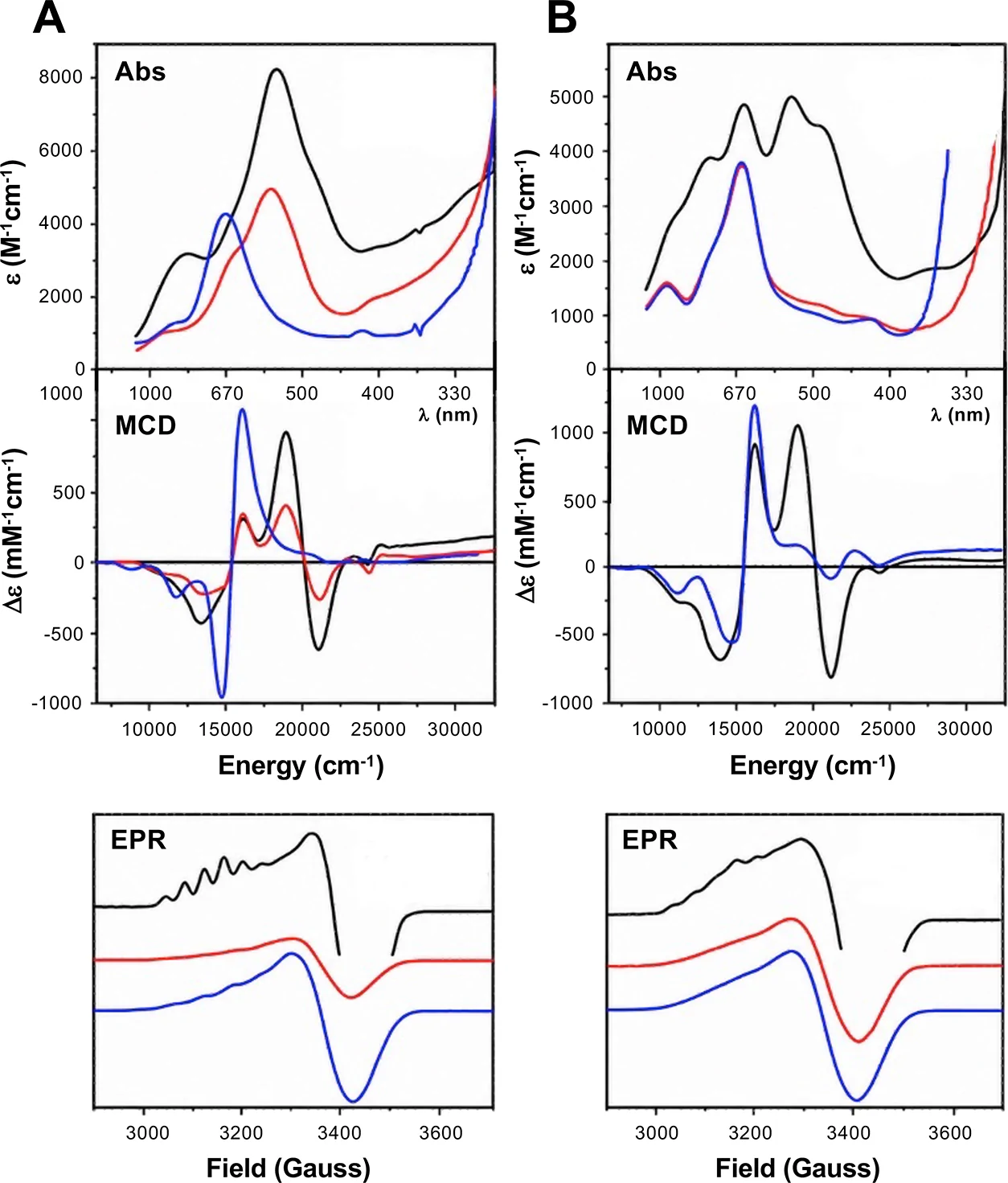
Spectroscopic comparison of purified *P. stutzeri* N_2_OR with CuZ cluster mainly as CuZ [4Cu:2S] (**A**) or CuZ* [4Cu:1S] (**B**). Room temperature absorption, low-temperature MCD (7 T, 5 K), and low-temperature EPR (77 K) in the fully oxidized state (black), ascorbate-reduced state (red), and dithionite-reduced state (blue). The oxidation state of CuA and CuZ centers in these spectra are described in Table S1 to facilitate its interpretation. Reproduced from.^3^ Copyright 2014 American Chemical Society.

The reduced CuA center is diamagnetic (*S* = 0) and spectroscopically silent because both copper ions are in the Cu^1+^ oxidation state, with 3d^10^ configurations. In contrast, the one-electron oxidized CuA center is, as mentioned before, a fully delocalized mixed-valence [Cu^1.5+^:Cu^1.5+^] species. This assignment was supported by multifrequency EPR spectroscopy, whereas UV–visible near-infrared absorption, circular dichroism (CD) and low-temperature magnetic circular dichroism (MCD) spectroscopies allowed the characteristic optical transitions to be assigned to Scys → Cu ligand-to-metal charge-transfer bands and to a low-energy intervalence transition associated with electron delocalization within the binuclear CuA center.^29,31,40,41^

The nature of the second copper center proved considerably more enigmatic. The absorption spectrum of N_2_OR in the reduced state, in which the CuA center is diamagnetic and does not contribute, exhibited an absorption band centered near 650 nm and has a broad, poorly resolved four-line EPR hyperfine pattern, with *g*-values at 2.16 and 2.04 (Figure 1A).^30,31,42,43^ These features were distinct from previously characterized copper centers. Together with low-temperature MCD data and considering the copper content determined for the isolated enzyme (four coppers per polypeptide chain), these observations led to the proposal of a second multinuclear copper center. Before structural information became available, this site was interpreted as a binuclear copper center able to access different oxidation states and most likely coordinated, at least in part, by histidine imidazole ligands.^30,42^

Redox titrations further showed that this copper center could be oxidized and reduced independently of CuA center, supporting its assignment as the catalytic site for N_2_O reduction. Because its structure was unknown, it was designated CuZ cluster,^30,42^ where ″Z″ simply reflected its distinction from the already established CuA and CuB centers and from the type 1, type 2 and type 3 copper center classifications.^3,44^

The structure of CuZ cluster was first revealed by the X-ray structure of N_2_OR from *M. nauticus*,^39^ which showed that the catalytic site was not binuclear, as initially proposed, but instead consisted of four copper ions arranged in a compact tetranuclear cluster. In the original structural model, the central bridging ligand was assigned as an oxygen-derived ligand. However, this assignment was difficult to reconcile with biochemical and spectroscopic evidence for sulfur coordination at the catalytic site.

Elemental analysis together with resonance Raman spectroscopy of isotopically labeled N_2_OR from *P. pantotrophus*, indicated that CuZ contained one acid-labile sulfur ligand.^45^ While resonance Raman spectroscopy studies on N_2_OR from *P. stutzeri* provided further evidence that sulfur contributes directly to the vibrational and electronic structure of the CuZ cluster.^46^ Re-examination of the structure of N_2_OR from *M. nauticus*, together with the determination of the *P. denitrificans* N_2_OR structure and sulfur analysis of both enzymes, established that CuZ cluster is a tetranuclear copper cluster bridged by an inorganic μ_4_-sulfido ligand and arranged in a distorted tetrahedral geometry (Figure 2A).^47^

**Figure 2:**
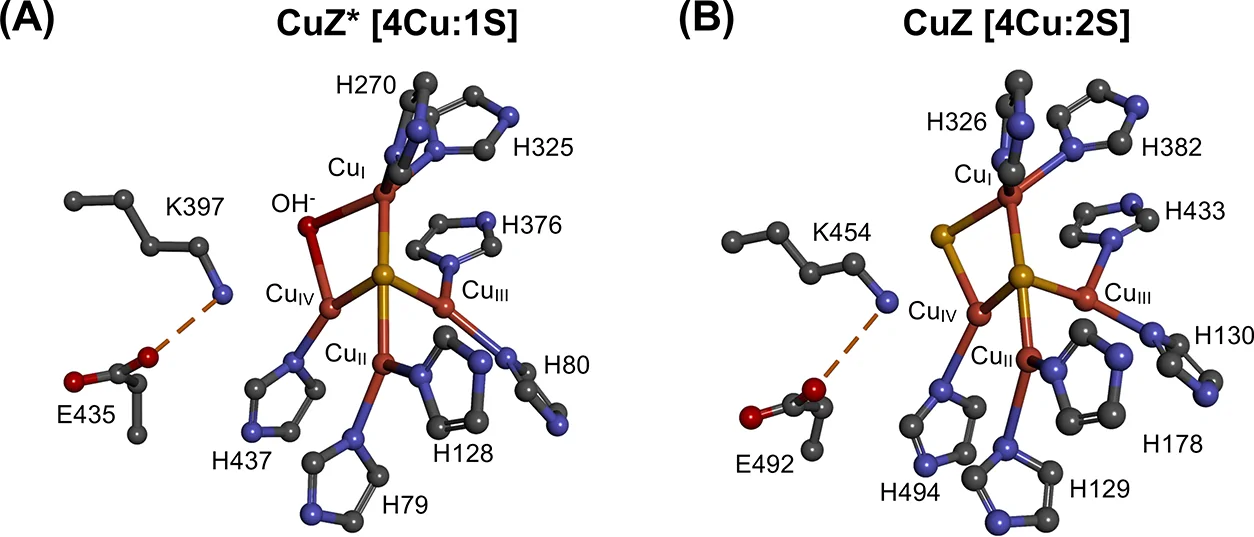
Different forms of CuZ cluster. (A) CuZ* [4Cu:1S] in *P. denitrificans* N_2_OR and (B) CuZ [4Cu:2S] in *P. stutzeri* N_2_OR. Figures were prepared with Biovia Discovery Studio using PDB ID 1FWX for *P. denitrificans* N_2_OR and PDB ID 3SBP for *P. stutzeri* N_2_OR. Color scheme for the atoms: carbon in gray, Cu in orange, N in blue, S in yellow, and O in red.

However, the structural assignment of CuZ as a tetranuclear copper-sulfide cluster did not fully resolve the heterogeneity observed among N_2_OR preparations. Spectroscopic studies of enzymes isolated under strictly anoxic conditions or exposed to oxygen during purification showed that the catalytic center could exist in distinct forms with different optical, EPR and redox properties (Figure 1).^42,48,49^ Studies of *P. pantotrophus* N_2_OR showed that limited oxygen exposure altered the redox behavior of the catalytic center, generating a form that was described as “redox-inactive” and spectroscopically related, but not identical, to the reduced redox-active CuZ species.^49^

The tetranuclear cluster observed in the first crystal structures of N_2_OR from *M. nauticus* and *P. denitrificans* is now generally assigned to the CuZ* form, corresponding to a CuZ* [4Cu:1S] cluster.^39,47^ In contrast, the structure of N_2_OR from *P. stutzeri*, obtained from the enzyme isolated under anoxic conditions, revealed a second sulfido ligand and established the existence of the [4Cu:2S] form, designated CuZ (Figure 2B).^50^ This finding provided a structural basis for the long-recognized spectroscopic heterogeneity of the CuZ cluster and showed that the catalytic site of N_2_OR can occur as two related but chemically distinct tetranuclear copper–sulfur clusters, CuZ* [4Cu:1S] and CuZ [4Cu:2S] (Figure 2).

In the CuZ* [4Cu:1S] form, the four copper ions are coordinated by conserved histidine residues from the N-terminal β-propeller domain. Each copper ion is coordinated by two histidine residues, with exception of Cu_IV_, which is coordinated by only one histidine imidazole. Coordination occurs through either the N^ε2^ atom of the imidazole ring, as observed for His80, His128, His270, His325 and His376, or through the N^δ1^ atom, as observed for His79 and His437 (numbering of the residues is according to the mature primary sequence of *P. denitrificans* N_2_OR).^47^ In addition to the μ_4_-sulfido ligand, a solvent-derived ligand bridges Cu_I_ and Cu_IV_ (Figure 2A). The coordination environment of the CuZ [4Cu:2S] form, is similar, but the solvent-derived Cu_I_-Cu_IV_ bridge is replaced by an additional sulfido ligand, giving rise to a second μ-bridging sulfur atom between Cu_I_ and Cu_IV_ ^50,51^ (Figure 2B).

The presence of four copper ions in the CuZ cluster gives rise to considerable redox flexibility and, in principle, allows the cluster to access five formal oxidation levels, ranging from the fully reduced [4Cu^1+^] state to the fully oxidized [4Cu^2+^] state (Scheme 1). However, only a subset of these formal oxidation states has been experimentally observed. In isolated N_2_OR, the CuZ cluster has been detected predominantly in mixed-valence forms corresponding to [2Cu^2+^:2Cu^1+^] and [1Cu^2+^:3Cu^1+^], whereas the fully reduced [4Cu^1+^] state can be produced *in vitro* upon prolonged chemical reduction.^52^ The ability of the cluster to distribute electrons over four copper ions, together with the presence or absence of the second sulfido ligand in CuZ [4Cu:2S] and CuZ* [4Cu:1S], provides the basis for its distinctive spectroscopic signatures and strongly influences its catalytic properties. These aspects are discussed in detail in the following section.

**Scheme 1:**
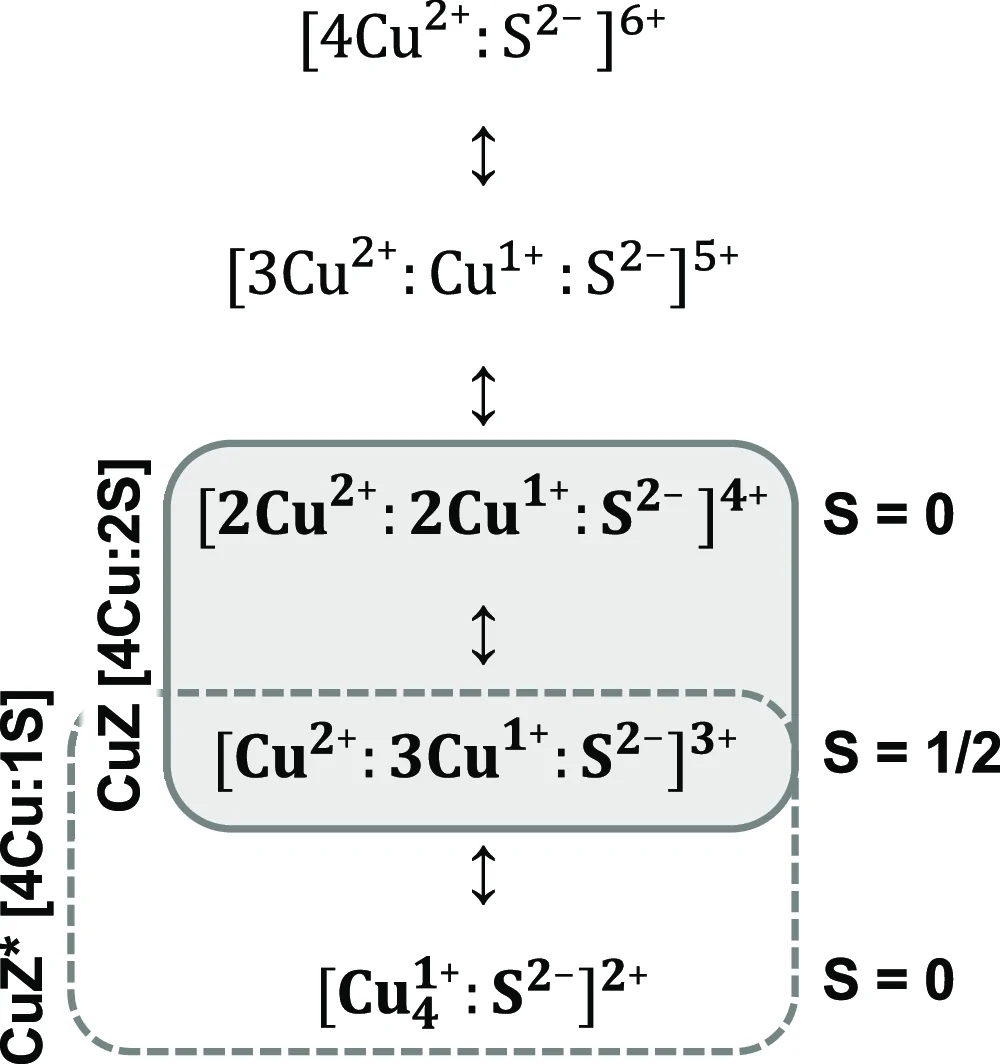
Possible Oxidation States of the Core CuZ Cluster and Spin States^a^

a The oxidation states in bold are the ones observed so far in N_2_OR.

## Spectroscopic, Redox and Catalytic Properties of CuZ Forms

The structural distinction between CuZ* [4Cu:1S] and CuZ [4Cu:2S] is reflected in their redox, spectroscopic and catalytic properties. In CuZ* [4Cu:1S], variable field MCD in combination with Cu K-edge XAS established the formal oxidation state of the cluster as [1Cu^2+^:3Cu^1+^].^53^ This form exhibits an absorption band centered at approximately 640 nm, with an ε_640 nm_ of around 3.5 mM^–1^cm^–1^, and an axial EPR signal characterized by *g*_∥_ = 2.16 and *g*_⊥_ ≈ 2.04, with a broad and poorly resolved hyperfine pattern in the parallel region (Figure 1B).^22,30,34,42,43,45,53^

A combination of Q-band EPR, low-temperature MCD, resonance Raman spectroscopy and density functional theory (DFT) calculations has been used to rationalize the unusual electronic structure of CuZ* [4Cu:1S].^46,53–56^ These studies showed that the spin density (*S*_Total_ = 1/2) is not localized on a single copper center but is distributed over the cluster and has predominantly Cu d_*x* ^2^–y^2^_ character. DFT calculations based on the crystallographic structure indicated that the spin density is mainly shared between Cu_I_, Cu_IV_ and the μ_4_-sulfido ligand, with calculated contributions of approximately 26% on Cu_I_, 13% on Cu_IV_ and 29% on the bridging sulfido ligand.^53^ This delocalization provides a molecular explanation for the broad EPR signal and for the intense sulfur-to-copper charge-transfer band observed in the visible region.^3^

Attention has been given to the nature of the Cu_I_-Cu_IV_ edge in CuZ* [4Cu:1S], since this position differentiates CuZ* [4Cu:1S] from the later identified CuZ [4Cu:2S] form. Several DFT models were evaluated, including structures containing a bridging water molecule, a water molecule terminally bound to Cu_I_, a bridging hydroxo, a hydroxo ligand bound to Cu_I_ and hydrogen-bonded to a protonated lysine residue.^53,55,56^ The model that best reproduced the experimental spectroscopic properties placed a hydroxo ligand at the Cu_I_-Cu_IV_ edge, positioned closer to Cu_I_ than to Cu_IV_, with calculated Cu_I_–OH and Cu_IV_–OH distances of approximately 2.00 and 2.09 Å, respectively.^56,57^ In this model, Lys397 and Glu435 (residue numbering according to the mature primary sequence of *P. denitrificans* N_2_OR) are hydrogen-bonded to each other,^56^ and the protonation state of Lys397 influences the position of the Cu_I_-Cu_IV_ hydroxide edge ligand.^56^

The redox chemistry of CuZ* [4Cu:1S] is also distinctive. The [1Cu^2+^:3Cu^1+^] state is not oxidizable (chemically or electrochemically)^43,58–60^ nor readily reduced by sodium dithionite alone (Scheme 1), despite the low formal reduction potential of sodium dithionite (*E*°′ of −471 mV vs SHE, at pH 7.0).^61^ However, prolonged incubation with reduced methyl viologen or benzyl viologen generates the fully reduced [4Cu^1+^] state^55^ (using either sodium dithionite or Zn-reduced viologens, C. Luciano, Master thesis NOVA FCT, unpublished data). Because this conversion behaves as an irreversible activation process under potentiometric conditions,^62^ the reduction potential of the [1Cu^2+^:3Cu^1+^]/[4Cu^1+^] couple has not been directly determined.^58^

In contrast to CuZ* [4Cu:1S], the CuZ [4Cu:2S] has been observed in two mixed-valence oxidation states, [2Cu^2+^:2Cu^1+^] and [1Cu^2+^:3Cu^1+^] (Scheme 1), with a reported reduction potential of approximately +60 mV at pH 7.5.^49,58^ The fully reduced [4Cu^1+^] state of CuZ [4Cu:2S] has not been obtained, even under strongly reducing *in vitro* conditions.^57,58^ The more oxidized [2Cu^2+^:2Cu^1+^] state is characterized by an absorption band around 550 nm, with an extinction coefficient of approximately 5.0 mM^–1^cm^–1^, whereas the [1Cu^2+^:3Cu^1+^] state displays a lower-energy absorption band centered near 670 nm, with extinction coefficient values in the range of 3.0–4.4 mM^–1^cm^–1^ (Figure 1A).^49,50,57,59,60^

The EPR properties of CuZ [4Cu:2S] are also oxidation-state dependent, with the [2Cu^2+^:2Cu^1+^] state being EPR-silent and was shown by MCD to be diamagnetic, whereas the [1Cu^2+^:3Cu^1+^] state is paramagnetic.^57^ The latter exhibits an axial EPR signal (*g*_∥_ > *g*_⊥_ > 2.0) and five evenly spaced hyperfine lines in the g_∥_ region (Figure 1A).^57,59^ The signal was interpreted using three equivalent hyperfine contributions, with *A*_∥_ and *A*_⊥_ values of approximately 5.6 mT and 2 mT, respectively,^57^ indicating that the ground-state spin is distributed over three copper centers and has predominantly *Cu d*_*x* ^2^‑*y* ^2^_ character.^57^ In a simple first-order treatment, coupling of a single *S* = 1/2 spin equally to three Cu nuclei (*I* = 3/2) would give rise to a 10-line hyperfine pattern. However, experimentally only five lines are resolved in the *g*_∥_ region. DFT calculations further suggested that the unpaired spin is distributed over Cu_I_, Cu_II_ and Cu_IV_, with calculated contributions of approximately 17%, 11% and 10%, respectively, while substantial spin density is also located on the μ_4_-sulfido ligand, the μ_2_-SH^–^ ligand and Cu_III_, with contributions of approximately 34%, 16% and 6%, respectively.^57^

Resonance Raman spectroscopy, combined with DFT calculations, showed that the protonation state of the Cu_I_-Cu_IV_ edge ligand in CuZ [4Cu:2S] depends on the oxidation state of the cluster. In the [2Cu^2+^:2Cu^1+^] state, the Cu_I_-Cu_IV_ edge is occupied by a sulfido ligand, assigned as μ_2_-S^2–^, with an apparent p*K*_a_ ≤ 3.^63^ In contrast, in the [1Cu^2+^:3Cu^1+^] state, the edge ligand is protonated and is best described as a hydrosulfido, μ_2_-SH^–^, with an apparent p*K*_a_ ≥ 11.^63^ This assignment was supported by the observation of D_2_O-sensitive vibrational modes attributed to S–H bending modes.^63^ These data indicate that reduction of CuZ [4Cu:2S] is accompanied by protonation of the Cu_I_-Cu_IV_ edge sulfur ligand, providing spectroscopic evidence for coupling between electron redistribution and proton transfer at the catalytic site.

The distinct redox and spectroscopic properties of CuZ [4Cu:2S] and CuZ* [4Cu:1S] are directly linked to their catalytic behavior. The need for activation of N_2_OR was inferred from the discrepancy between the high N_2_O-reducing activity observed in crude cell extracts and the low specific activity typically measured for purified enzymes.

Crude extracts reduce exogenously supplied N_2_O at rates of approximately 48–72 μmol N_2_O·min^–1^·mg_N2OR_^–1^.^12,64–66^ In contrast, isolated N_2_OR preparations containing variable proportions of CuZ* [4Cu:1S] and CuZ [4Cu:2S] usually display much lower specific activities, typically between 1 and 10 μmol N_2_O·min^–1^·mg_N2OR_^–1^.^12,17,20,49,60^ These low activities were therefore assigned to an inactive or “unready” state of the enzyme.^25,67^ A notable exception is the N_2_OR from *W. succinogenes*, for which a specific activity of approximately 160 μmol N_2_O·min^–1^·mg_N2OR_^–1^ has been reported, suggesting that this enzyme may not require an activation as other enzymes.^24,68,69^

Activation of N_2_OR occurs upon prolonged incubation with reduced viologens.^52,70^ Early activation experiments showed that the increase in catalytic activity correlates with the reduction of CuZ* [4Cu:1S] from the [1Cu^2+^:3Cu^1+^] state to the fully reduced [4Cu^1+^] state.^52^ This conversion is accompanied by disappearance of the CuZ* [4Cu:1S] EPR signal, consistent with formation of a diamagnetic species in which all four copper ions are in a d^10^ electronic configuration.^52^ The activation process is slow, with a rate constant of approximately 1.2 × 10^–3^ s^–1^ at pH 7.0, and is therefore too slow to correspond to a step within the catalytic turnover cycle.^70,71^ Once formed, the fully reduced CuZ* [4Cu:1S] [4Cu^1+^] state displays a high catalytic competence, with a turnover number estimated at approximately 320 s^–1^.^71^ By contrast, for CuZ [4Cu:2S], the catalytically active state has been assigned to the [1Cu^2+^:3Cu^1+^] oxidation state, but the reported turnover number is much lower, of approximately 0.6 h^–1^.^71^ These observations indicate that the different structural forms of the CuZ cluster are not functionally equivalent and that the identity of the bridging ligand at the Cu_I_-Cu_IV_ edge has a major influence on catalytic activity. Whether the fully reduced [4Cu^1+^] state of CuZ* [4Cu:1S] is reached under physiological conditions has remained a point of discussion among research groups working on N_2_OR. Although this question has not yet been definitively resolved, kinetic comparisons between isolated enzyme and whole-cell N_2_O reduction provide important insight.

For N_2_OR from *M. nauticus*, kinetic assays performed with reduced methyl viologen as electron donor gave a maximum specific activity of approximately 200 μmol N_2_O·min^–1^·mg_N2OR_^–1^ and a *K*_M_ for N_2_O of 18 μM for the enzyme containing CuZ* [4Cu:1S] in the fully reduced [4Cu^1+^] oxidation state.^72^ A similar apparent affinity constant was obtained in whole-cell assays measuring the reduction of exogenously added N_2_O.^66^ In addition, when the yield of N_2_OR purification was taken into account, the maximum specific activity determined for the isolated enzyme was sufficient to explain the high N_2_O-reduction rates observed in cells grown under nitrate-respiring conditions.^66^ These results support the hypothesis that the CuZ* [4Cu:1S] [4Cu^1+^] oxidation state may correspond to a catalytically ready form of the enzyme *in vivo*.

Additional support for the functional relevance of the Cu_I_-Cu_IV_ edge came from structural studies of ligand binding. Iodide, which has been proposed to inhibit N_2_OR, was shown to bind to the CuZ cluster at the Cu_I_-Cu_IV_ edge.^73^ This is the same region where a solvent-derived ligand was modeled in the structure of *A. cycloclastes* N_2_OR containing CuZ* [4Cu:1S] in the [1Cu^2+^:3Cu^1+^] oxidation state^73^ (Figure 3A). This observation provided a structural basis for computational studies of N_2_O binding to the CuZ cluster. DFT calculations subsequently identified a μ-1,3-N_2_O coordination mode across the Cu_I_-Cu_IV_ edge of fully reduced CuZ* [4Cu:1S] [4Cu^1+^] as a favorable substrate-binding geometry^3,52,54,74^ (Figure 3B). Nevertheless, direct experimental evidence for substrate coordination to CuZ* [4Cu:1S] remains limited.

**Figure 3:**
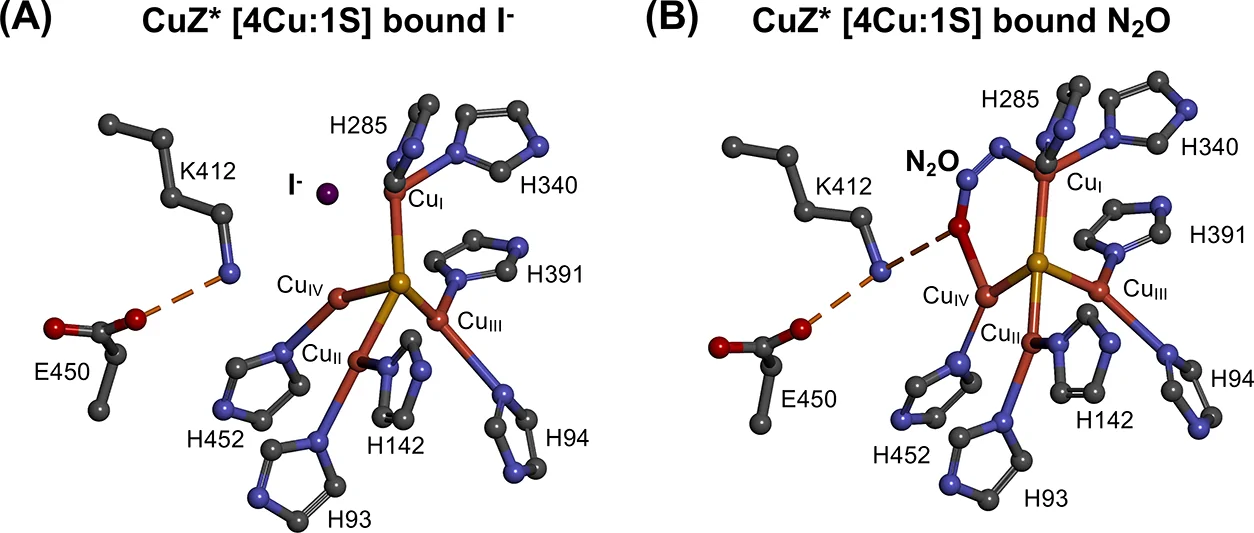
Structure of CuZ cluster of *A. cycloclastes* N_2_OR. (A) Coordination of iodide-bound CuZ* [4Cu:1S] and (B) of N_2_O-bound CuZ* [4Cu:1S] (N_2_O was modeled into the structure with data from^52^). Figures were prepared with Biovia Discovery Studio using PDB ID 2IWK for *A. cycloclastes* inhibitor-bound N_2_OR and PDB ID 2IWF for *A. cycloclastes* N_2_O-bound N_2_OR. Color scheme for the atoms: carbon in gray, Cu in orange, N in blue, S in yellow, O in red and I in violet.

An alternative attempt to visualize substrate binding was made using crystals of *P. stutzeri* N_2_OR containing CuZ [4Cu:2S] in the [2Cu^2+^:2Cu^1+^] oxidation state, which were pressurized with N_2_O.^50^ In this structure, N_2_O was placed above the CuZ cluster, but the distance to the copper ions was too long to be considered coordinating any of the copper ions.^50^

Moreover, given that CuZ [4Cu:2S] in the [2Cu^2+^:2Cu^1+^] oxidation state was shown not to react with N_2_O, the observed N_2_O molecule is unlikely to represent a catalytically relevant substrate-bound state.^71^

Taken together, the available biochemical, spectroscopic and kinetic data indicate that, although CuZ* [4Cu:1S] is frequently observed in enzymes isolated under conditions that may perturb the catalytic center, such as exposure to oxygen or growth of the cells at acidic pH,^49,59,60^ it is the only form of the CuZ cluster that has so far been associated with high specific activity following activation to the fully reduced [4Cu^1+^] state (see next section). Thus, the available evidence does not allow CuZ* [4Cu:1S] to be classified simply as a physiologically irrelevant form and CuZ [4Cu:2S] as the physiologically relevant form. Rather, catalytic competence appears to depend on the oxidation state and coordination environment at the Cu_I_–Cu_IV_ edge, while it remains unresolved to what extent the CuZ forms observed after isolation reflect physiologically populated states or are influenced by growth and purification conditions. Another important unresolved question is how CuZ [4Cu:2S] is converted *in vivo* into a catalytically competent state and whether this process requires displacement or modification of the μ_2_-sulfido ligand at the Cu_I_-Cu_IV_ edge. It also remains to be established whether NosR (see next section), or another membrane-associated protein complex, participates directly in activation of the catalytic center and in maintaining N_2_OR in an active state during turnover.

## Catalytic Cycle of CuZ* Cluster

A catalytic cycle for CuZ* [4Cu:1S] has been proposed based on structural, spectroscopic, kinetic and computational studies (Figure 4). In this mechanism, catalysis begins from the fully reduced [4Cu^1+^] state of CuZ* [4Cu:1S] (intermediate 1), which reacts with N_2_O to generate a substrate-bound intermediate 2.^74^ Initial binding occurs through the terminal nitrogen atom of N_2_O to Cu_I_ in a linear configuration, followed by N–O bond elongation and rearrangement to a μ-1,3-N_2_O binding mode across the Cu_I_-Cu_IV_ edge, with an N–N–O angle of approximately 139° (Figure 3B). In this intermediate, the oxygen atom of N_2_O is hydrogen-bonded to protonated Lys397, positioning the substrate for proton-coupled N–O bond cleavage.^52,75,76^

**Figure 4:**
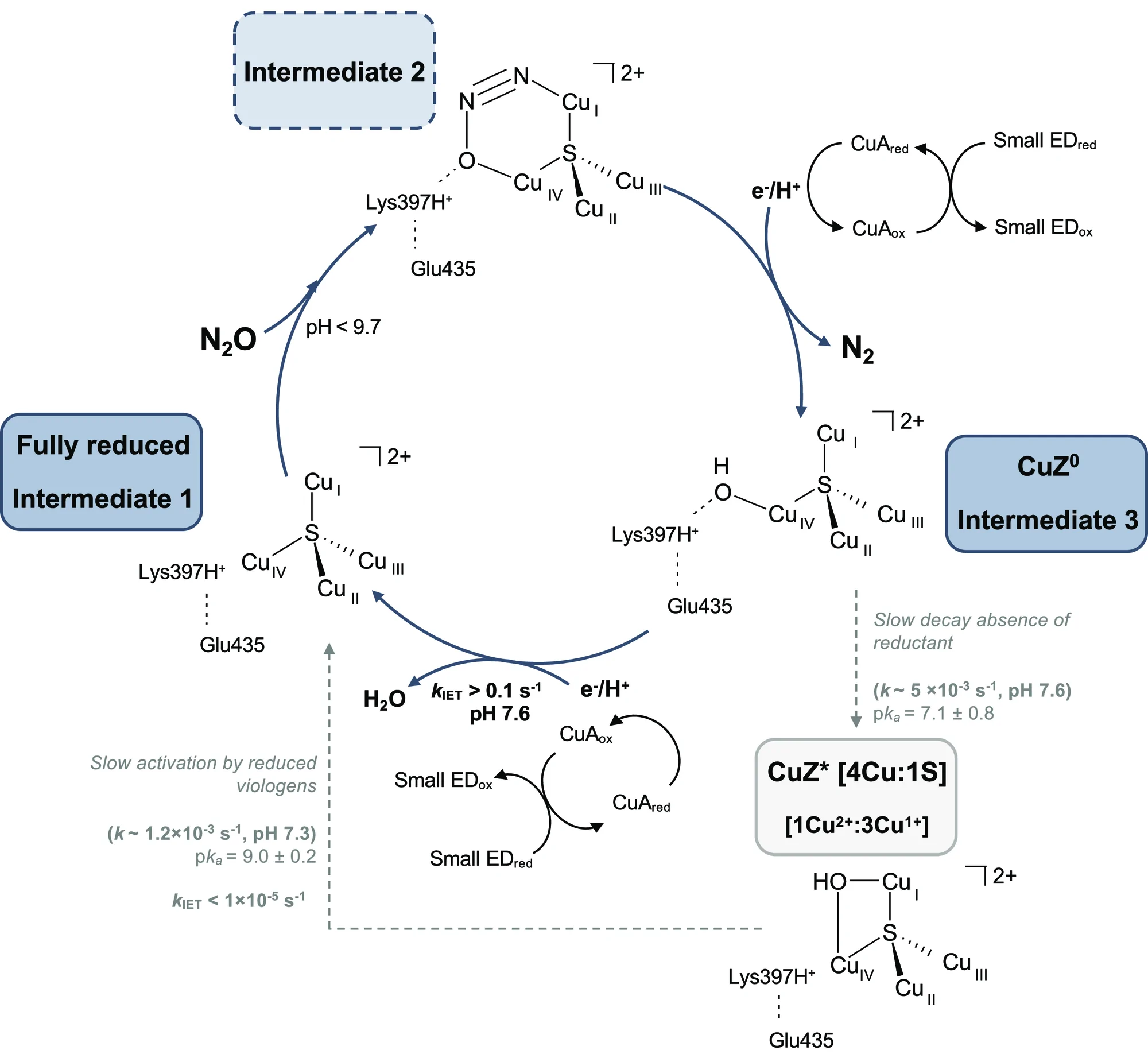
Catalytic cycle of N_2_O reduction by N_2_OR with CuZ* [4Cu:1S]. The intermediate species were proposed based on DFT calculations and on experimental data on CuZ^0^. In the scheme is represented the reductive activation by reduced viologens, which is not part of the catalytic cycle. Residues are numbered according to *P. denitrificans* N_2_OR mature primary sequence. ED = electron donor.

Release of N_2_ requires transfer of two electrons from the fully reduced CuZ* [4Cu:1S], with Cu_IV_ acting as a key conduit for electron delivery and all four copper ions contributing to the electronic reorganization of the active site.^74^ Cleavage of the N–O bond is proposed to involve transfer of one electron coupled to proton transfer from Lys397 to the substrate oxygen atom, generating a Cu_IV_-bound hydroxo intermediate. A second electron-transfer step then promotes cleavage of the Cu_I_-N interaction and release of N_2_, and reprotonation of Lys397 by solvent, together with electron transfer from CuA center, produces the CuZ^0^ intermediate, in which protonated Lys397 hydrogen-bonds to the Cu_IV_-bound hydroxo ligand and to Glu435, thereby stabilizing the oxidized state.^74^ The catalytic cycle is completed by intramolecular electron transfer (IET) through the CuA center, with a reported rate constant *k*_IET_ > 0.1 s^–1^.^74^

The CuZ^0^ intermediate was first trapped by reacting fully reduced CuZ* [4Cu:1S] [4Cu^1+^] with an equimolar amount of N_2_O.^62^ This reaction generated a short-lived species with an absorption band with a maximum at approximately 680 nm, with an extinction coefficient of around 2.0 mM^–1^ cm^–1^, together with absorption bands at 480, 540, and 800 nm attributed to oxidized CuA center.^62,74^

The EPR spectrum of CuZ^0^ is axial and, after subtraction of the CuA center contribution, can be simulated with *g*_∥_ = 2.177 and *g*_⊥_ = 2.05.^62^ Six hyperfine features are resolved in the *g*_∥_ region. Simulation of the spectrum using two equivalent copper hyperfine contributions gave *A*_∥_ = 4.2 mT, consistent with delocalization of the unpaired spin over two copper nuclei.^74^ As observed for CuZ [4Cu:2S], fewer hyperfine features are experimentally resolved than expected from a simple first-order treatment, for which coupling of a single *S* = 1/2 spin equally to two Cu nuclei (*I* = 3/2) would give a seven-line pattern. Resonance Raman and MCD spectroscopies further supported assignment of CuZ^0^ as having a [1Cu^2+^:3Cu^1+^] oxidation state and that its coordination environment differs from that of CuZ* [4Cu:1S] in the same oxidation state.^74^ DFT calculations suggested that CuZ^0^ contains a hydroxo ligand terminally coordinating Cu_IV_ and hydrogen-bonded to a protonated Lys397, resulting in a more even distribution of spin density between Cu_I_ and Cu_IV_ and no bridging ligand.^74^

Importantly, formation of CuZ^0^ is associated with catalytically competent N_2_OR,^62^ and CuZ^0^ can be reduced back to the fully reduced [4Cu^1+^] state using sodium ascorbate, a physiologically relevant artificial electron donor.^74^ This reduction proceeds through rapid intramolecular electron transfer, indicating that the reduction potential of CuZ^0^ must be above 0 mV and therefore compatible with reduction by periplasmic electron donors.^74^ This behavior parallels that observed in multicopper oxidases, in which the isolated resting oxidized state is reduced too slowly to account for catalytic turnover, whereas the native intermediate is reduced at catalytically relevant rates. Similarly, the rapid reduction of CuZ^0^ compared with the resting CuZ* [4Cu:1S] in the [1Cu^2+^:3Cu^1+^] oxidation state supports CuZ^0^ as the catalytically relevant oxidized form of the CuZ cluster and highlights that states accumulating upon enzyme isolation do not necessarily correspond to those populated during turnover.^77^

## Assembly of the CuZ Cluster in N_2_OR

The CuZ cluster is positioned at the center of the N-terminal seven-bladed β-propeller domain of N_2_OR. It is approximately 40 Å from the CuA center within the same subunit (Figure 5A), a distance that does not allow efficient intramolecular electron transfer, but only around 10 Å from the CuA center of the other subunit.^47,50,51,73^ This spatial arrangement makes N_2_OR a functional dimer, in which electron transfer occurs between the CuA center of one subunit and the CuZ cluster of the other (Figure 5A). In contrast to the CuA center, which is bound to the C-terminal β-barrel domain and lies close to the protein surface, CuZ cluster is buried and not solvent exposed. This location may explain why attempts to reconstitute CuZ cluster either *in vitro* or during heterologous production of N_2_OR in microorganisms that do not naturally encode this enzyme have been unsuccessful.^78^ This indicates that assembly of the CuZ cluster requires dedicated accessory proteins. N_2_OR is encoded by the *nosZ* gene, which is organized in a conserved operon together with genes encoding proteins involved in CuZ maturation (Figure 5B).

**Figure 5:**
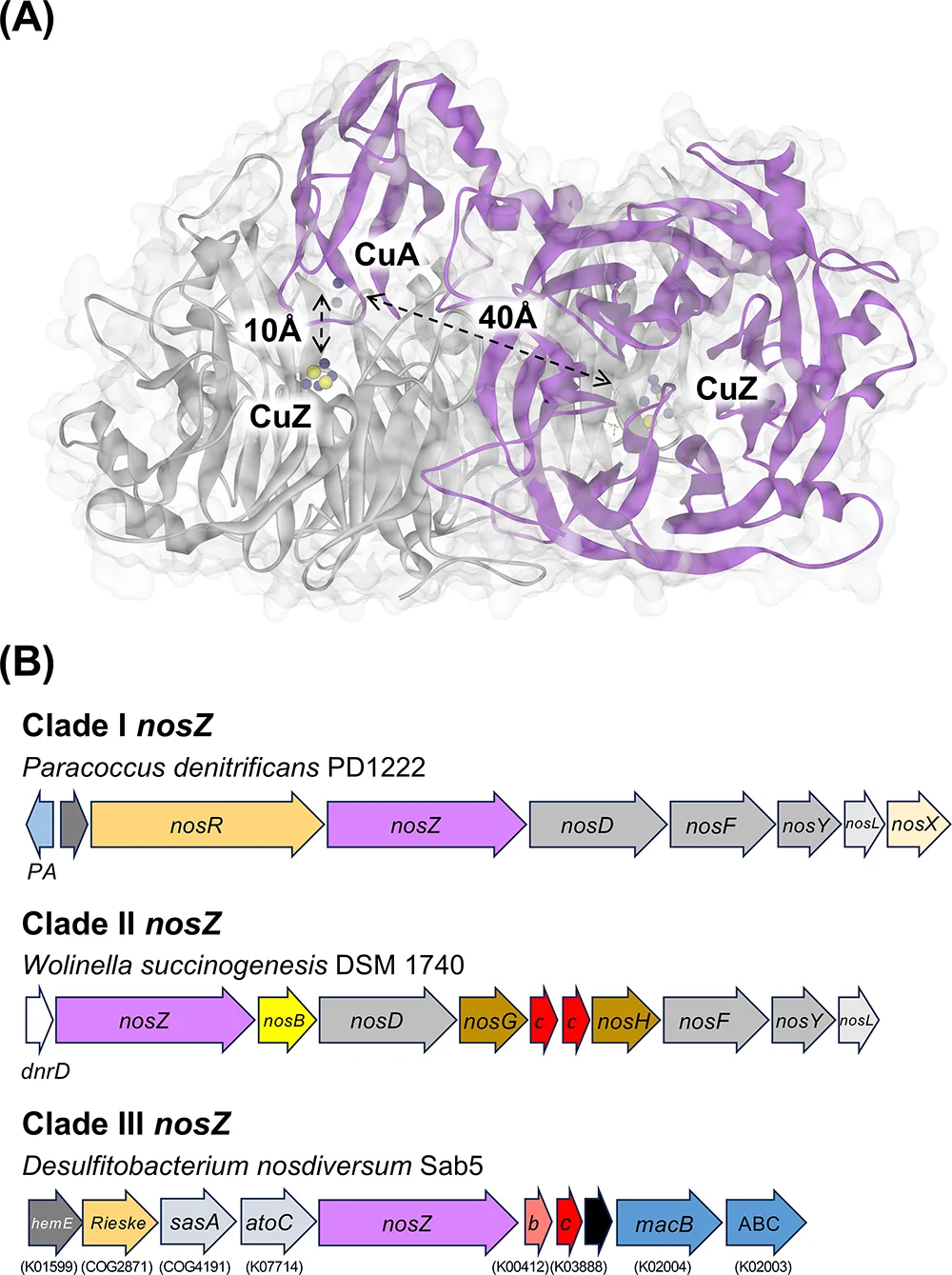
N_2_OR functional homodimer and genomic organization of *nosZ* gene cluster. (A) Structure of *P. stutzeri* N_2_OR functional homodimer. The backbone of one monomer is represented with the identified secondary structure colored in violet and the other monomer is similarly represented in gray. CuA and CuZ clusters are represented by spheres, in which the copper atoms are colored in blue and sulfur atoms in yellow. The distances between CuA and CuZ clusters of the two monomers are given. Figure was prepared with Biovia Discovery Studio using PDB ID 3SBP. (B) Genomic organization of *nosZ* gene cluster of representatives of each N_2_OR clade. Functional annotation of Clade III genes is given as described in.^79^

Although N_2_OR catalyzes the final step of the denitrification pathway, the *nosZ* gene has also been identified in nondenitrifying microorganisms and is present in approximately 12% of sequenced bacterial and archaeal genomes.^80^ Phylogenetic analyses based on protein sequence and structure have led to the identification of three clades, designated Clade I, II and III,^79–81^ which also differ in the gene composition of the *nosZ* operon (Figure 5B). Clade III differs from Clade I and II by the absence of the conserved *nosDFYL* genes, which have been shown to be essential to the assembly of the CuZ cluster in the N_2_OR polypeptide chain.^82,83^ Earlier studies had already shown that disruption or absence of *nosD*, *nosY* or *nosF* resulted in N_2_OR depleted of the CuZ cluster.^34,84–86^

NosL is a 18 kDa copper chaperone composed of two structurally independent domains with a ββαβ topology and is membrane-anchored through a lipidated cysteine residue,^83,87–89^ and shown to be required for CuZ assembly under physiologically relevant copper concentrations.^83,89^ This lipoprotein binds Cu^1+^ specifically in a 1:1 stoichiometry with attomolar affinity,^83,87^ as part of a heterodinuclear metal cluster formed together with a zinc ion.^89^ The zinc ion has been proposed to be required for the functional integrity and stability of NosL.^89^

NosDFY is an ATP-binding cassette-type complex, NosDF_2_Y_2_, that promotes copper insertion into N_2_OR by functioning as a Cu^1+^-transfer system from NosL to N_2_OR.^90^ NosF_2_Y_2_ forms a membrane-associated heterodimer, whereas NosD protrudes into the periplasm as an elongated β-helix, structurally similar to carbohydrate-binding and glycoside hydrolase protein family, as previously predicted from primary sequence analysis.^91,92^ NosF had previously been isolated and shown to have Mg^2+^-dependent ATPase activity with *K*_M_ values of 3 mM and 10 mM for ATP and GTP hydrolysis, respectively.^93^ Cryo-EM structures provided snapshots of the NosDFY mechanism. In the absence of ATP, NosDFY forms a complex with NosL, allowing transfer of a copper ion to NosD. ATP binding induces a conformational change that promotes release of NosL and recruitment of N_2_OR for copper uptake. Subsequent ATP hydrolysis releases N_2_OR from the complex, allowing another cycle of copper delivery to occur.^90^ It has long been hypothesized that NosDFY would be involved in sulfur transport to the CuZ cluster,^85,91^ but how this process occurs remains unclear, and no evidence for sulfur transport was observed in the available cryo-EM maps.

A supermolecular complex involving N_2_OR and other enzymes of the denitrification pathway has been identified, revealing the presence of NosDFY but also NosR, a protein encoded in the *nosZ* operon of Clade I N_2_ORs.^94,95^ NosR is an Fe/S- and FMN-containing membrane protein required for whole-cell N_2_O reduction.^96,97^ It has been proposed to be involved in maintaining N_2_OR in an active state,^92^ consistent with the observation that N_2_OR isolated from *nosR* knockout strains had CuZ cluster as CuZ* [4Cu:1S].^96^ NosR has also been proposed to participate in electron transfer from the quinol pool to N_2_OR,^98^ either directly or through additional soluble electron carriers.

Although *nosR* is absent from Clade II *nosZ* clusters, these encode *c*-type cytochromes and *nosHG*, which are homologues to genes encoding quinol dehydrogenase NapHG,^99^ that have been proposed to constitute an electron-transfer pathway from menaquinol to N_2_OR.^65,69^ Thus, in Clade II microorganisms, NosHG and associated cytochromes may fulfill a role functionally analogous to that proposed for NosR in Clade I systems.

As mentioned above, Clade III N_2_ORs have a distinct *nosZ* gene cluster organization.^79^ This gene cluster encode *b*-type cytochromes and two ATP-binding cassette transporter components (one being a MacB-type transporter),^79^ which may have roles analogous to those of NosDFY in N_2_OR maturation and/or electron-transfer systems. Future studies on Clade III N_2_OR will be required to define the functions of these proteins, to determine which form of CuZ cluster is present in these enzymes, and to clarify whether differences in active-site assembly, second-sphere coordination or electron delivery pathway contribute to the whole-cell N_2_O-reduction activity observed under acidic conditions, at which Clade I N_2_OR generally show very low or no activity.^59,100^

## Chemical Modeling CuZ Cluster

The unusual structure and reactivity of the CuZ cluster have stimulated the development of synthetic copper–sulfur complexes that reproduce selected structural, electronic or functional features of N_2_OR. The challenge is considerable, because the biological site combines several demanding properties in a single active center: a tetranuclear copper core, sulfido coordination, accessible oxidation states, electron delocalization, a reactive Cu_I_-Cu_IV_ edge, and a second coordination sphere capable of controlling proton delivery and substrate activation. Consequently, synthetic model compounds have not aimed simply to reproduce the complete enzyme active site, but rather to isolate individual contributions from copper nuclearity, sulfide coordination, ligand donor type, oxidation state and secondary-sphere interactions.^101^

Initial model systems showed that reactivity toward N_2_O is not restricted to tetranuclear copper clusters. Tolman and co-workers reported a mixed-valent tricopper-disulfido cluster, formulated as a [Cu^2+^:Cu^1+^:Cu^1+^] species, that reacts with N_2_O to generate N_2_. Computational analysis proposed an alternative binding mode in which N_2_O interacts through its oxygen atom in a μ-1,1 fashion at a dicopper fragment,^102^ distinct from the μ-1,3 binding mode proposed for the N_2_OR CuZ* [4Cu:1S].^52,76^ These studies were important because they provided functional precedence for copper–sulfur-mediated N_2_O reduction, while also illustrating that model reactivity may follow pathways different from those proposed for the enzyme.

Other lower-nuclearity copper-sulfur complexes have been developed to probe the minimum structural requirements for N_2_O activation. Tricopper sulfido clusters supported by N-heterocyclic carbene, phosphine or nitrogen-rich ligands provided useful platforms for understanding how sulfido coordination and copper nuclearity can be controlled synthetically.^103–105^ In particular, nitrogen-rich and coordinatively unsaturated copper-sulfide clusters represented a step toward the histidine-rich environment of the native CuZ cluster. However, these systems did not reproduce its tetranuclear [Cu_4_S] architecture and therefore are better regarded as partial structural models or mechanistic probes rather than direct mimics of CuZ cluster.

Dicopper sulfide complexes have also contributed to understanding N_2_O reactivity. A dissymmetric mixed-valent {Cu_2_S} complex containing labile ligands was shown to reduce N_2_O to N_2_, whereas related complexes with more symmetric or saturated coordination environments were inactive or less reactive.^106,107^ These results support the idea that the presence of an accessible and electronically differentiated dicopper edge is important for N_2_O activation. This is conceptually relevant to CuZ* [4Cu:1S], where the Cu_I_-Cu_IV_ edge has been proposed to provide the substrate-binding and N–O bond-cleavage site. In a complementary study, Bagherzadeh and Mankad showed that reaction of a dicopper­(I) μ-sulfide complex with N_2_O resulted in oxidation of the sulfide ligand, highlighting a possible additional role of the tetranuclearity of CuZ cluster in protecting the sulfide ligand from oxidative degradation during N_2_O activation.^108^

More structurally faithful models emerged with tetranuclear [Cu_4_(μ_4_-S)] clusters. Yam and co-workers reported a phosphine-supported [Cu_4_(μ_4_-S)] cluster before the crystallographic structure of N_2_OR had been determined.^109^ Although this complex reproduced the tetranuclear copper-sulfide core, displaying *C*_2_ symmetry with two short (∼2.9/2.6 Å) and two long Cu–Cu distances (∼3.1/3.4 Å) and a sulfur-centered geometry with τ_4_ = 0.59 (τ_4_ is a structural parameter used to quantify the geometry of four-coordinate centers, with values approaching 0 corresponding to a more square-planar geometry and values approaching 1 corresponding to a more tetrahedral geometry), it was stabilized in a fully reduced [4Cu^1+^] state and was not reactive toward N_2_O.^109–112^ Thus, reproducing the {Cu_4_S} core and overall geometry alone was insufficient to reproduce the redox and catalytic properties of the enzymatic center, emphasizing the importance of the ligand environment and electronic structure of the cluster.

Subsequent phosphine-supported [Cu_4_S] models allowed further exploration of cluster assembly, redox tuning and inhibitor binding. Mankad and co-workers showed that {Cu_4_S} and {Cu_3_S} clusters could be assembled using diphosphine ligands, and that iodide binding could perturb the nuclearity of the cluster, emphasizing the importance of the protein scaffold in maintaining the integrity of the biological CuZ cluster.^101,104^

A systematic study of related [Cu_4_(μ_4_-S)] models further showed that ligand electronics and steric protection strongly influence cluster assembly, stability and redox accessibility.^113^ Neutral phosphine donors were found to stabilize [Cu_4_(μ_4_-S)] clusters in the [4Cu^1+^] state, whereas anionic nitrogen-donor ligands enabled access to higher oxidation states relevant to CuZ cluster chemistry.^113^ These observations emphasize that the supporting ligand framework controls not only formation of the tetranuclear copper-sulfide core, but also the oxidation states that can be stabilized.

A major advance was achieved with the synthesis of nitrogen-supported [Cu_4_(μ_4_-S)] clusters using amidinate ligands,^63^ which provided a closer analogue of the histidine-ligated environment of CuZ cluster, with near-perfect *C*_*2V*_ symmetry, short Cu–Cu distances of 2.4 Å and long Cu–Cu distances of 3.0 Å, and a sulfur geometry characterized by a τ_4_ of 0.76.^63^ This complex reproduced important structural and redox features of the CuZ/CuZ* family and could be reduced from a [2Cu^2+^:2Cu^1+^] to a [1Cu^2+^:3Cu^1+^] oxidation state, with further irreversible reduction leading to a fully reduced [4Cu^1+^] form. In the oxidized state, it absorbs in the visible region with a band centered at 561 nm and a shoulder at 470 nm and is EPR silent.^114^ The spectroscopic and redox properties point to a similarity to CuZ [4Cu:2S] rather than CuZ* [4Cu:1S]. Nevertheless, the [1Cu^2+^:3Cu^1+^] oxidation state of this {Cu_4_S} cluster was subsequently characterized as a structural and functional model of CuZ* [4Cu:1S], exhibiting an *S* = 1/2 ground state and reactivity toward N_2_O.^114^ It was then proposed that the oxidized and reduced forms of this complex and the fully reduced complex proposed by Yam et al. would complete a synthetic cycle for N_2_O reduction.

This system therefore represented an important progression from structural mimicry toward functional modeling, demonstrating that a nitrogen-rich ligand environment can support both biologically relevant oxidation states and N_2_O reactivity. At the same time, the absence of the protein secondary coordination sphere limits its ability to reproduce the full complexity of the CuZ cluster.

The electronic role of the μ_4_-sulfido ligand in this synthetic [Cu_4_(μ_4_-S)] system was subsequently examined by combined experimental and computational approaches.^115^ Cu K-edge and S K-edge XAS, supported by DFT calculations, showed substantial participation of the μ_4_-sulfido ligand in the redox-active molecular orbital of the highly covalent [Cu_4_(μ_4_-S)] core.^115^ These results are particularly relevant to the enzymatic CuZ/CuZ* cluster, because they show that the bridging sulfido ligand should not be viewed as a purely structural ligand, but as an active participant in the electronic structure and redox chemistry required for N_2_O reduction.

Later studies highlighted the importance of the secondary coordination sphere. A {Cu_4_S} cluster in the [4Cu^1+^] oxidation state was shown to mediate the 2H^+^/2e^–^ reduction of N_2_O to N_2_ and H_2_O in the presence of an external reductant, with second-sphere N–H groups acting as proton donors.^116^ The reactivity depended strongly on both the primary coordination sphere and second-sphere hydrogen-bonding and proton-relay effects, indicating that CuZ models require more than the correct metal-sulfide core to reproduce enzymatic behavior. These results parallel the proposed role of Lys397 and the surrounding protein matrix in positioning substrate and controlling proton delivery at the Cu_I_-Cu_IV_ edge of the N_2_OR CuZ cluster.

More recent work has further refined the relationship between structure, electronic distribution and reactivity in {Cu_4_S} models. Meyer and co-workers isolated {Cu_4_(μ_4_-S)} clusters using a flexible hexadentate macrocyclic ligand system L that provides two binding pockets and NHC ligation for the Cu ions, in the [1Cu^2+^:3Cu^1+^] and [4Cu^1+^] oxidation states. These complexes were characterized by combining X-ray diffraction, NMR, UV–visible spectroscopy, EPR, Cu/S K-edge X-ray emission spectroscopy, Cu K-edge XAS and time-dependent DFT calculations to define how geometric and electronic structures change with oxidation state.^117^ These studies showed a planarization of the core {Cu_4_S} upon oxidation that gives rise to π* interactions between Cu 3d_*yz*_/3d_*xz*_ orbitals and the μ_4_-S 3p_*z*_ orbital. In the [1Cu^2+^:3Cu^1+^] oxidation state, the calculated spin density is almost equally distributed between the μ_4_-S ligand and the Cu ions (in total), with the metal contribution arising predominantly from two Cu ions located opposite each other, similarly to that proposed for the CuZ^0^ intermediate species.^117^ These studies provide valuable comparisons with the fully reduced [4Cu^1+^] and [1Cu^2+^:3Cu^1+^] oxidation states of CuZ* [4Cu:1S] and raise the question of whether redox changes at the CuZ cluster may be coupled to protein-scaffold strain, histidine-ligand reorientation, and hydrogen-bond rearrangements, allowing the {Cu_4_S} core to undergo functionally relevant geometric and electronic changes during N_2_O reduction.

In parallel, Mankad showed that very closely related triazenide-supported {Cu_4_S} clusters can display divergent reactivity: instead of promoting N_2_O reduction to N_2_ and H_2_O, these systems mediate N_2_O disproportionation.^118^ This observation is particularly important because it demonstrates that subtle ligand changes can redirect the reaction pathway even when the overall {Cu_4_S} core is preserved.

Most recently, site differentiation within tetracopper sulfide clusters has emerged as a key factor in biomimetic N_2_O activation. Using diffraction anomalous fine structure measurements and computational modeling, Mankad and co-workers showed that hydrogen-bond-induced structural distortion correlates with Cu-site differentiation and enhanced N_2_O reduction activity.^119^ This provides a synthetic analogue for a central principle of the enzyme: although CuZ is a homometallic cluster, the individual copper ions are not chemically equivalent during catalysis. Differentiation of copper sites, polarization of specific Cu–Cu edges and second-sphere control may therefore be essential features for productive N_2_O activation.^119^

In conclusion, synthetic CuZ models have progressed from structural analogues to increasingly sophisticated systems that reproduce oxidation states, spectroscopic signatures and functional N_2_O reactivity. These studies show that tetranuclearity alone is not sufficient for enzymatic-like function. Instead, catalytic behavior depends on the combined effects of copper nuclearity, sulfide coordination, ligand donor type, cluster distortion, redox accessibility, proton delivery and stabilization by the surrounding matrix. Although no synthetic system yet fully reproduces the efficiency, reversibility and robustness of N_2_OR, these models provide essential platforms for testing mechanistic hypotheses and for guiding the design of future N_2_O-reduction catalysts.

## Concluding Remarks and Future Perspectives

The increasing environmental relevance of N_2_O emissions has reinforced the need to understand, preserve and eventually exploit the biological process responsible for its conversion to N_2_. N_2_OR catalyzes the final step of denitrification, reducing N_2_O to N_2_, and therefore represents a central enzyme for both the nitrogen cycle and strategies aimed at mitigating N_2_O emissions from anthropogenic and natural sources. In this context, detailed knowledge of the catalytic CuZ cluster, including its maturation, activation and susceptibility to environmental stress, is essential for understanding why N_2_O reduction fails under certain conditions and how this process might be enhanced in soils, sediments, wastewater treatment systems and engineered microbial platforms.

The chemical complexity of the CuZ cluster reflects the intrinsic difficulty of N_2_O reduction. Although the reaction is thermodynamically favorable, activation of the N–O bond requires a highly specialized catalytic site capable of controlling electron and proton delivery. The structure and electronic properties of the different forms of CuZ cluster have been elucidated over the past years but several important questions remain unanswered.

Structural characterization of fully reduced CuZ* [4Cu:1S] and of the CuZ^0^ intermediate at high resolution would be particularly valuable, as these states are directly connected to catalysis but remain incompletely defined. Such information would clarify the coordination environment of the active cluster, the position of proton-delivery residues, and the structural changes that might accompany N_2_O binding and reduction.

Another central open question is whether N_2_OR requires activation *in vivo* and, if so, how the active form is generated and maintained during turnover. The isolation of N_2_OR containing CuZ [4Cu:2S], a form with low specific activity, has raised the possibility that this form of the cluster may represent a resting, protected or incompletely activated state when substrate, electrons or appropriate cellular partners are unavailable. However, the mechanism by which this form could be converted into a highly active state remains unknown. It is unclear whether activation requires displacement, protonation or rearrangement of the μ_2_-bridging sulfido ligand at the Cu_I_-Cu_IV_ edge, and/or whether other protein-mediated processes are involved. Accessory proteins, including NosR or membrane-associated redox complexes, may contribute to this process, but their direct role in CuZ activation and catalytic maintenance still requires experimental validation.

Alongside biochemical and spectroscopic investigations, inorganic chemistry has made important contributions to our understanding of CuZ cluster. Synthetic CuZ models provide a valuable complement to enzymatic studies, as they enable the individual roles of copper nuclearity, sulfide coordination, ligand environment, and oxidation state to be examined in the absence of the protein matrix. However, no synthetic models reproducing the {Cu_4_S_2_} core have yet been reported, and existing systems have not provided structural insight into substrate-bound states of the active site. In addition, achieving long-term stability, reversible access to multiple oxidation states, efficient proton-coupled reactivity, and sustained catalytic turnover remains a significant challenge for future studies.

Future advances in structural biology, spectroscopy, enzymology, microbial physiology and synthetic inorganic chemistry will be essential to resolve these questions. A more complete understanding of CuZ assembly, activation and catalysis will not only clarify how nature overcomes the kinetic barrier of N_2_O reduction but may also support the development of strategies to enhance biological N_2_O removal and to design new catalysts for N_2_O conversion. In this way, the study of N_2_OR and its unique tetranuclear copper-sulfide active site continues to connect fundamental bioinorganic chemistry with one of the most pressing environmental challenges associated with the nitrogen cycle.

## Supplementary Material



## Notes

Table describing the oxidation states of CuA and CuZ sites in the different oxidation states of N_2_O reductase (PDF)
