# Supplementary material for: Tetranuclear Copper Cluster of N2O Reductase: A Distinctive Copper–Sulfur Metallocluster
Source: Inorg Chem. 2026 Oct 6;65(41):24083–96. doi: 10.1021/acs.inorgchem.6c04238 (PMC13685413; doi:10.1021/acs.inorgchem.6c04238)
Supplement: Supplementary file 1 [file ic6c04238_si_001.pdf]

Supplementary Information

The Tetranuclear Copper Cluster of N_2O Reductase: A Distinctive Copper-Sulfur Metallocluster

Sofia R. Pauleta[1,2,*], José J. G. Moura[3] and Isabel Moura[3]
[1]Microbial Stress Lab, UCIBIO – Applied Molecular Biosciences Unit, Departamento de Química, Faculdade de Ciências e Tecnologia, Universidade NOVA de Lisboa, 2829-516 Caparica, Portugal
[2]Associate Laboratory i4HB - Institute for Health and Bioeconomy, Faculdade de Ciências e Tecnologia, Universidade NOVA de Lisboa, 2829-516 Caparica, Portugal
[3]LAQV, REQUIMTE, Departamento de Química, Faculdade de Ciências e Tecnologia, Universidade Nova de Lisboa, 2829-516 Caparica, Portugal

Corresponding Author
Sofia R. Pauleta
Email: sofia.pauleta@fct.unl.pt

Table S1. Oxidation states of the copper centers of N_2OR in the fully oxidized, ascorbate-reduced and dithionite-reduced state.

	N_2OR with mainly CuZ		N_2OR with mainly CuZ*	
**Oxidation state of the enzyme**	**CuA**	**CuZ cluster**	**CuA**	**CuZ cluster**
Oxidized	$[1Cu^{1.5+}:1Cu^{1.5+}]$	$[2Cu^{2+}:2Cu^{1+}]$	$[1Cu^{1.5+}:1Cu^{1.5+}]$	$[1Cu^{2+}:3Cu^{1+}]$
Ascorbate-reduced	$[1Cu^{1+}:1Cu^{1+}]$	$[2Cu^{2+}:2Cu^{1+}]$	$[1Cu^{1+}:1Cu^{1+}]$	$[1Cu^{2+}:3Cu^{1+}]$
Dithionite-reduced	$[1Cu^{1+}:1Cu^{1+}]$	$[1Cu^{2+}:3Cu^{1+}]$	$[1Cu^{1+}:1Cu^{1+}]$	$[1Cu^{2+}:3Cu^{1+}]$
